# Flavonoids and Furanocoumarins Involved in Drug Interactions

**DOI:** 10.3390/molecules30081676

**Published:** 2025-04-09

**Authors:** Sabine Berteina-Raboin

**Affiliations:** Institut de Chimie Organique et Analytique (ICOA), Université d’Orléans UMR-CNRS 7311, BP 6759, Rue de Chartres, CEDEX 2, 45067 Orléans, France; sabine.berteina-raboin@univ-orleans.fr; Tel.: +33-238-494-856

**Keywords:** drug interactions, furanocoumarins, anthocyanins, flavonoids, coumarins

## Abstract

Drug interactions can have significant consequences for public health, especially given the growing importance of readily available dietary supplements. The same applies to the consumption of fruit and fruit juices, which are often praised for their health benefits, but which can generate drug interactions. These are well known and documented in the case of grapefruit, which should not be taken with certain medications. Grapefruit contains flavonoids and furanocoumarins, which are responsible for various interactions with the cytochrome P450 enzyme system. However, for young children and the elderly, fruit juices are often used to facilitate treatment. This review examines commonly used fruit juices, particularly from citrus, apple, and red fruits, and discusses potential interactions, disadvantages, and advantages, as well as the chemical structures involved in interactions with cytochromes P-450, P-glycoprotein, and organic anion transporter polypeptide (OATP), responsible for sometimes dangerous changes in bioavailability or potential accumulation of drugs in the body.

## 1. Introduction

For the past 30–40 years, grapefruit and the compounds it contains (flavonoids, and especially furanocoumarins) have been studied for their interactions with certain drugs [1]. The interaction is not with the drug’s active ingredient, but with the biological processes involved in its metabolism [2]. Indeed, any plant substance taken concomitantly with a drug is likely to interact with it if the mechanisms involved in digestion and metabolism involve the same types of receptors [2,3]. The danger lies in the potential under- or overdosing of the drug, depending on the interaction, and the appearance of clinical consequences that can be serious [3,4,5], for example, the loss of therapeutic effect or the appearance of side effects. Furanocoumarins inhibit enzymes, which are thus neutralized and can no longer play their metabolic roles in the walls of the liver and intestine. While grapefruit is often implicated in scientific studies of drug interactions, these compounds are also found in other citrus fruits and vegetables. Nevertheless, grapefruit remains highly prized for other interesting effects, such as its contents of vitamin C, the antioxidant lycopene, beta-carotene, and others [1]. In 1989, the first scientific study showed its interaction with the metabolism of felodipine [2]. It should be noted that the compounds that appear to be responsible for sometimes serious clinical consequences [3] are present in much greater quantities in the white part of the fleshy grapefruit skin than in the juice. However, because the juice industry squeezes the fruit with the skin still attached, commercial fruit juices contain much higher levels of furanocoumarins than traditionally squeezed fresh fruit juices. The impact of consuming this fruit in association with certain drugs is therefore more or less significant, and difficult to quantify in daily consumption. It has also been shown that interactions are not only dose-dependent, but also person-dependent, and that it is extremely difficult to measure the potential impact of grapefruit consumption on patients suffering from certain pathologies that are more or less sensitive to these interactions [4]. Moreover, the presence or absence of grapefruit extracts is not always mentioned, making it difficult to avoid the risk of interactions [5]. These interactions arise from drug metabolism by cytochromes P450 (CYP). Cytochromes P450 represent a large family of hemoprotein-type enzymes. They share many common features, but are distinguished by their apoproteins [6,7].

Cytochromes P450 (CYPs) are divided into two major classes: the class of interest, involved in the metabolism of xenobiotics, and the class involved in the synthesis of various endogenous substances [6,7,8]. There are several CYPs, including six main types involved in oxidative drug metabolism, including CYP3A4, CYP2D6, CYP1A2, CYP2C8, CYP2C9, and CYP2C19 [7,9], but CYP3A is the main enzyme present in the liver and the most involved in drug metabolism. Some CYPs are also present in other organs, notably the intestine. Metabolism may therefore begin in the intestinal walls [2,10,11] before even reaching the liver, depending on how rapidly a given drug is absorbed. Drugs and their metabolites enter the various walls via membrane transporters. Compounds such as furanocoumarins and flavonoids inhibit these actions, thereby altering the bioavailability of orally administered drugs. This leads to potentially dangerous drug accumulation [12,13]. Of course, these deleterious interactions only apply to drugs administered via the enteral route, which is the most common method of administration.

**Figure 1:**
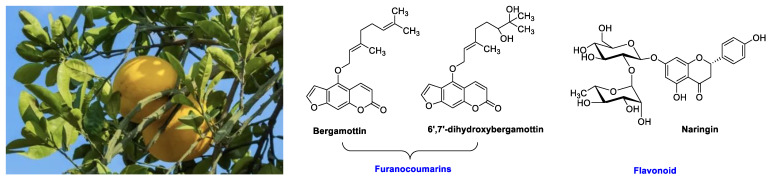
Main compounds involved in drug interactions found in grapefruit.

This interaction has been shown to occur mainly in metabolism by cytochromes P450 3A4 (CYP3A4). CYP3As are particularly important, as they are involved in a large number of metabolic processes. This enzymatic inhibition is linked to the presence, among others, of bergamottin and 6′,7′-dihydroxybergamottine, furanocoumarins present in significant quantities in grapefruit and listed as irreversible CYP3A4 inhibitors [3,11,14,15]. Mention should also be made of flavonoids, the most important of which in grapefruit is naringenin (Figure 1). Hepatic inhibition is also possible with high grapefruit consumption [16]. CYP3A4 inhibition persists for several days [17,18,19] and is dependent on furanocoumarin content. However, compositions are known to depend on many factors, from the terroir in which the fruit is grown, through storage conditions, to ripening and mode of consumption (fresh, pasteurized), meaning that many parameters need to be taken into account in order to explain the variable degrees of CYP3A4 inhibition reported [20].

The action of flavonoids, in particular naringin, on membrane transporters inhibits organic anion-transporting polypeptides (OATPs) [21]. This results in a transient decrease in the bioavailability of certain drugs [22,23,24,25]. A number of mechanisms favoring bioavailability [1] have also been identified via the inhibitory activities of furanocoumarins and flavonoids on P-glycoprotein (P-gp) [26]. Interactions have been demonstrated with other fruit juices, notably orange, bitter orange (Seville orange), bergamot, lime, tangelo, mandarin, certain red fruit, grape, and apple juices. To a lesser extent, these juices also inhibit CYP3A4 [27]. Similarly, at the OATP level, apple juice and orange juice could also increase the absorption of certain drugs [25], as they also contain flavonoids. These flavonoids could prove very useful in limiting the doses of drugs to be used, if it were possible to increase their bioavailability on demand. In this review, we will attempt to take stock of the chemical structures responsible for these inhibitions, in order to consider modifications that could be envisaged to reduce or increase their impacts.

## 2. Results and Discussion

There is extensive literature on the interactions between grapefruit juice and various drugs [1], including anti-cancer agents, anti-infectives, anti-lipemic agents, cardiovascular agents, CNS agents, gastrointestinal and urinary tract agents, etc. Grapefruit juice interferes with a large number of drugs; however, it is difficult to pinpoint the exact impacts of the compounds contained, so, as a precaution, it is recommended not to consume grapefruit juice when taking certain drugs, but what about other foods? D.G. Bailey et al. [28] showed that naringin was a major and selective clinical inhibitor of organic anion transporter polypeptide 1A2 (OATP1A2) in grapefruit juice. As mentioned in the introduction, this flavonoid has the opposite effect of furanocoumarins, helping to reduce drug absorption, particularly of fexofenadine, analyzed in this study, via grapefruit juice’s inhibition of the activity of an OATP family protein transporter. The balance between the inverse actions of furanocoumarins and flavonoids, therefore, makes it difficult to anticipate the bioavailability of drugs when consuming grapefruit juice.

Moreover, according to the same authors, orange and apple juices (1200 mL) also reduced absorption. Furanocoumarins and flavonoids reduced fexofenadine absorption by 28% and 23%, respectively, in these juices. It is not only fruit juices that are responsible for these metabolic problems. Other plant families, including those of certain vegetables, can also generate this type of interaction, even though cold-pressed juices are recommended for the benefits of their vitamin contents in cancer treatment. What about their impacts? In another example, pepper and its component piperine are thought to increase the bioavailability of certain drugs [29]. The same applies to turmeric, which is often sold with added piperine and taken as a dietary supplement for its anti-inflammatory action. The mechanisms of these effects are not fully defined, but it is thought that their effect on absorption is due in part to their inhibition of gastric emptying. An update on the various juices commonly consumed that may be involved in these drug interactions is listed here, together with the molecules that may be responsible for their effects. Only fruit types that have been studied in humans are mentioned here.

**Figure 2:**
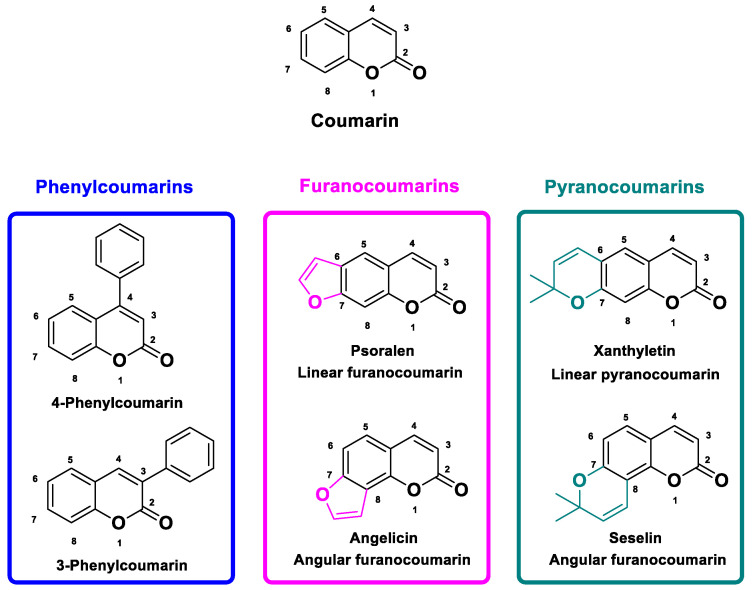
Typical classes of coumarins.

Coumarins represent an important group of phenolic compounds found in a wide variety of plants [30,31], listed and described in over 150 plant species [31]. All coumarin classes have a central structure composed of a benzo-α-pyrone, to which a furan ring can be attached, to yield furanocoumarins or a pyran ring that generate pyranocoumarins. Depending on the positions of these additional rings on the aromatic ring in positions 6, 7 or 7, 8, linear or angular derivatives are obtained. Of the structures to which phenylcoumarins can be added [30,31,32,33] (Figure 2), furanocoumarins, which are of interest to us here, have been referenced in several families [34], and are mainly found in the *Apiaceae* (parsnip, parsley, celery), *Fabaceae* (coronilla), *Moraceae* (fig), and, of course, *Rutaceae* (bergamot, citrus) families [30,31,33,35,36,37]. Furanocoumarins present in these plant families include psoralen, xanthotoxin, bergapten, angelicin, pimpinellin, sphondin, and isobergapten [38]. It is important to note, however, that the highest concentrations have been reported [39,40,41] in various *Apiaceae* species, such as parsnip (145 μg·g^−1^ FW) or parsley (112 μg·g^−1^ FW), where they occur in much higher concentrations compared with that in grapefruit juice (*Rutaceae*), the most targeted source of drug interactions, wherein the reported furanocoumarin concentration would only be of the order of 6 μg·g^−1^ FW [36,40,42], which is still much lower than that of some *Apiaceae* members.

### 2.1. Grapefruit and the General Citrus Family

**Figure 3:**
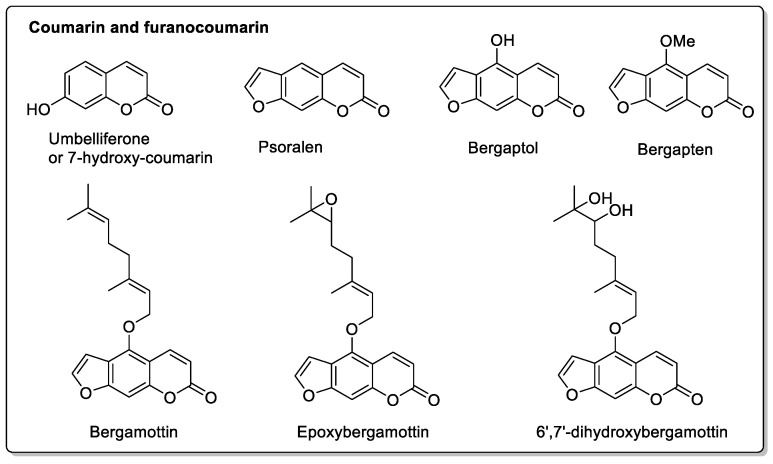
Various coumarin derivatives found in the citrus fruit family.

D. Black et al. [1] published an excellent review of drug interactions with grapefruit compounds. Mechanisms of action are discussed for a large number of drug classes. Here, we have mentioned potential pharmacokinetic and pharmacodynamic interactions, focusing mainly on the structures of the molecules involved. For compounds present in grapefruit (*Citrus × paradise*) and all citrus fruits, the biosynthetic pathway of target furanocoumarins starts with phenylalanine to generate umbelliferone or 7-hydroxy-coumarin. Depending on the enzyme, this then leads to linear or angular furanocoumarins. The proportion and presence of this or that enzyme in different citrus fruits generates one or the other, or both, which explains the modulation of the impacts on interactions depending on the citrus fruit studied (Figure 3) [43,44].

**Figure 4:**
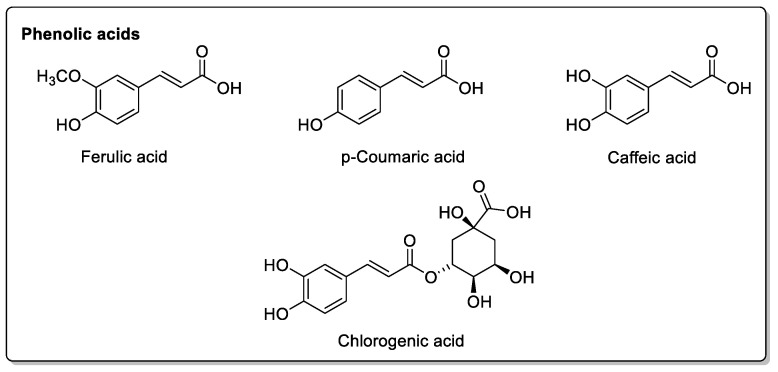
Various phenolic acids found in the citrus fruit family.

The phenolic derivatives found in citrus fruit are derived from caffeic acid, i.e., 3,4-dihydroxycinnamic acid (Figure 4).

**Figure 5:**
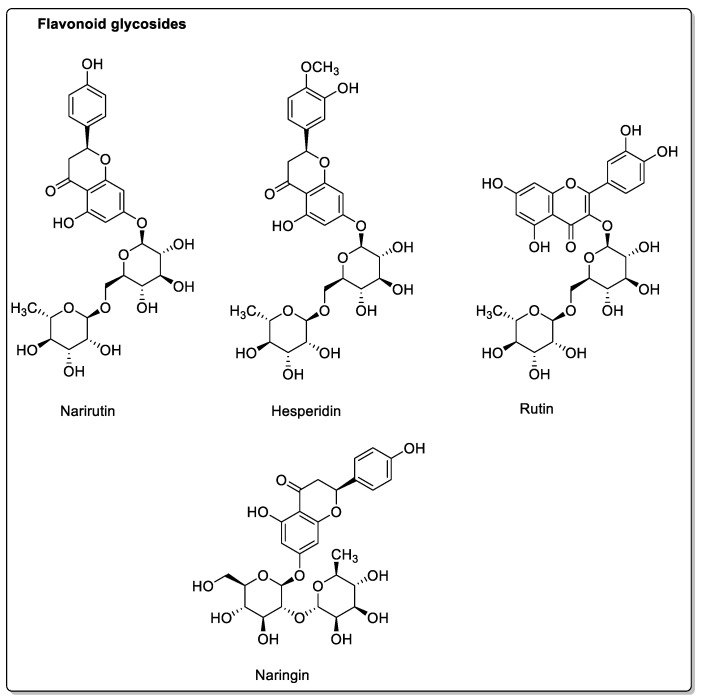
Various flavonoid glycosides found in the citrus fruit family.

Flavonoids include rutin, a glycosylated derivative of quercetin. It is an antioxidant found in many plants. Narutin is a derivative of (S)-naringenin, which also has antioxidant and anti-inflammatory properties. Naringin, also derived from (S)-naringenin, has antineoplastic and anti-inflammatory properties [45,46]. Hesperidin is a glycosidic derivative of hesperetin (Figure 5). All these derivatives of furanocoumarins, phenolic acids, and flavonoids, as well as flavonoid aglycones, are present in different citrus fruits in varying doses, and some compounds are even absent [47]. This probably explains the varying characteristics and interactions of these different citrus fruits.

#### 2.1.1. Orange

**Figure 6:**
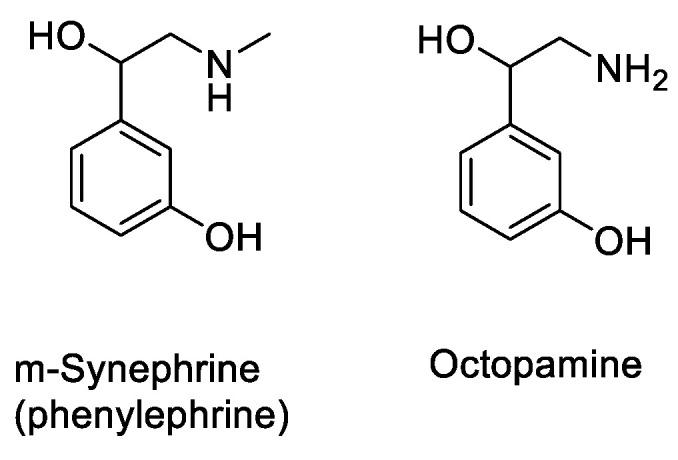
Additional compounds found in orange.

As for orange juice (*Valencia*), which is widely consumed, it seems that it can considerably reduce the bioavailability of certain drugs [1,48]. This interaction, as with many citrus fruits, could be due in part to modulation of intestinal wall transporters, but also to the acidity of the juices, which could ionize the active molecules, limiting their penetration capacity, as absorbable forms are often non-ionized. In addition to those mentioned above, among the molecules that inhibit OATP2B1 [21] and, therefore, absorption transporters, is hesperidin [49]. Hesperidin has a chemical structure very similar to that of naringin, but hesperidin does not produce complete inhibition of OATP1A2-mediated transport activity compared with naringin [28]. Seville orange juice (*Citrus aurantium*) may interact by inactivating intestinal CYP3A4, but does not alter P-gp concentration in enterocytes [50]. Depending on the citrus fruit, flavone glycoside composition differs. B.J. Gurley et al. [51] also studied the synephrine or octopamine content of Seville orange to assess its potential cardiovascular effects in humans (Figure 6).

M.H. Jin et al. [47] quantified constituent levels to measure its effects on systolic blood pressure and heart rate. They were able to show, in healthy volunteers, that no significant cardiovascular effects were observed, despite the presence of these compounds (Figure 6). Orange peel contains *p*-coumaric acid and ferulic acid (Figure 4), while the other two phenolic acids are absent. Flavonoid glycosides include narirutin and hesperidin (Figure 4), but not furanocoumarin. A notable variant, however, is Seville orange (bitter orange), in which all four phenolic acids are present, as well as narirutin and naringin, but not hesperidin, while one furanocoumarin (bergapten) is present (Figure 3).

#### 2.1.2. Lime

**Figure 7:**
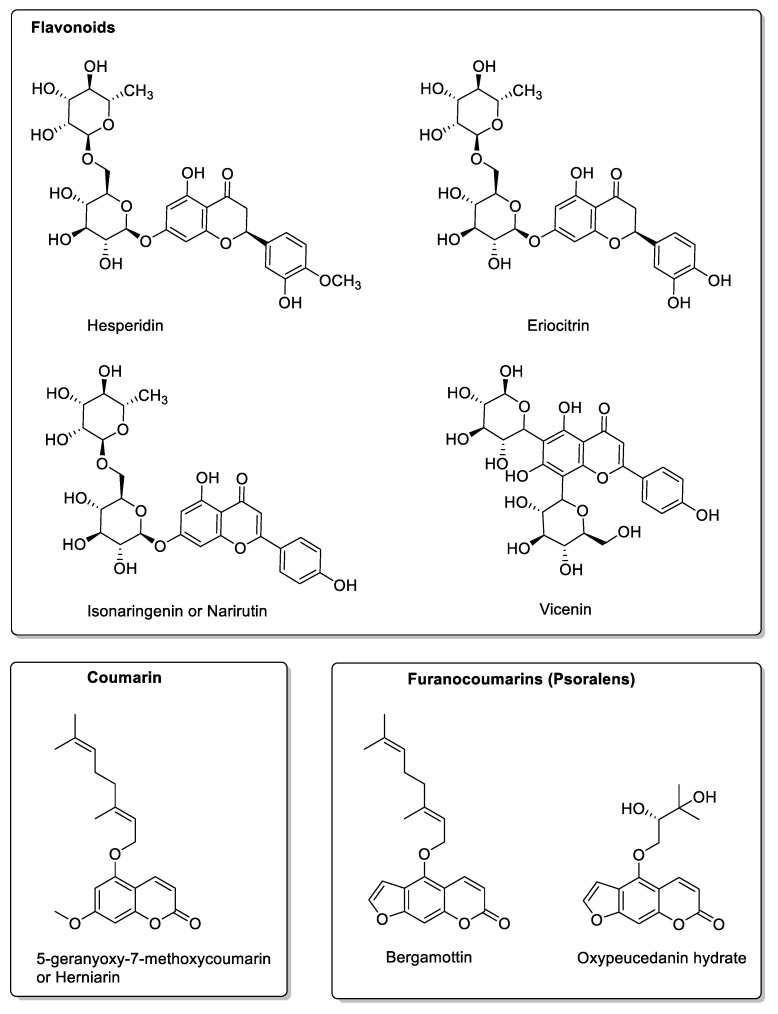
Additional compounds found in lime.

The interaction between lime juice (*Citrus aurantiifolia* and *Citrus latifolia*) and drugs is strongly linked to flavonoid compounds, but also to its high vitamin C content. Vitamin C’s antioxidant properties may play a role in the efficacy of certain drugs, such as antimalarials [52]. The health effects of vitamin C are manifold, including the inhibition of CYP3A4 [53] and CYP2C9, with concomitant action on OATP2B1, since the same flavonoids and polyphenols are found in grapefruit. Caffeic acid, p-coumaric acid, ferulic acid, rutin, narirutin, naringin, naringenin (flavonoid aglycone), hesperetin (flavonoid aglycone), bergapten, and bergamottin are found in citrus peel [47], and are, therefore, present in commercial fruit juices. L. Mondello et al. [54] demonstrated the presence of the main constituents in this juice by HPLC determination with UV detection. Their study was carried out on fruit from Calabria, Italy, and showed a high proportion of the flavonoids hesperidin and eriocitrin, as well as, to a lesser extent, isonaringenin, vicenin, and lucenin, in addition to lower proportions of coumarins (5-geranyloxy-7-methoxy-coumarin) and psoralens (bergamottin and oxypeucedanin hydrate) (Figure 7).

#### 2.1.3. Pomelo

**Figure 8:**
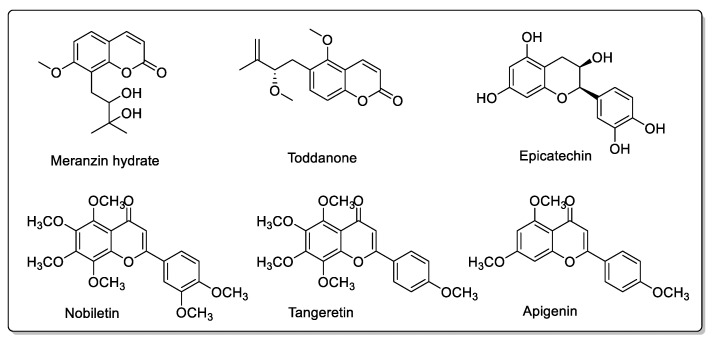
Various additional compounds found in *Citrus grandis* L.

Pomelo (*Citrus grandis* L. *Osbeck* or *Citrus maxima*) inhibits CYP2C9, CYP3A, and/or P-gp activity in the intestine. Drugs metabolized by CYP3A4 should be more bioavailable, but this effect is reduced by the action of pomelo juice on absorption transporters. Other physicochemical interactions may also be due to other components of pomelo juice [55]. In addition to being rich in vitamin C [56] *Citrus grandis*, like other citrus fruits, contains a variety of active compounds that are highly beneficial to health, but may also give rise to drug interactions [57]. These include rutin, meranzin hydrate, apigenin, tangeretin, nobiletin, and taddanone, as well as epicatechin [58], which is found in the peel of the pomelo, and also in green teas, where it does not appear to pose problems (Figure 5 and Figure 8). The pulp and juice of *Citrus grandis* L. contain phenolic acids such as caffeic acid, *p*-coumaric acid, and ferulic acid (Figure 4), as well as naringin, 5-geranyl-7-methoxycoumarin, and hesperidin, which are also present in other citrus fruits.

### 2.2. Fig

**Figure 9:**
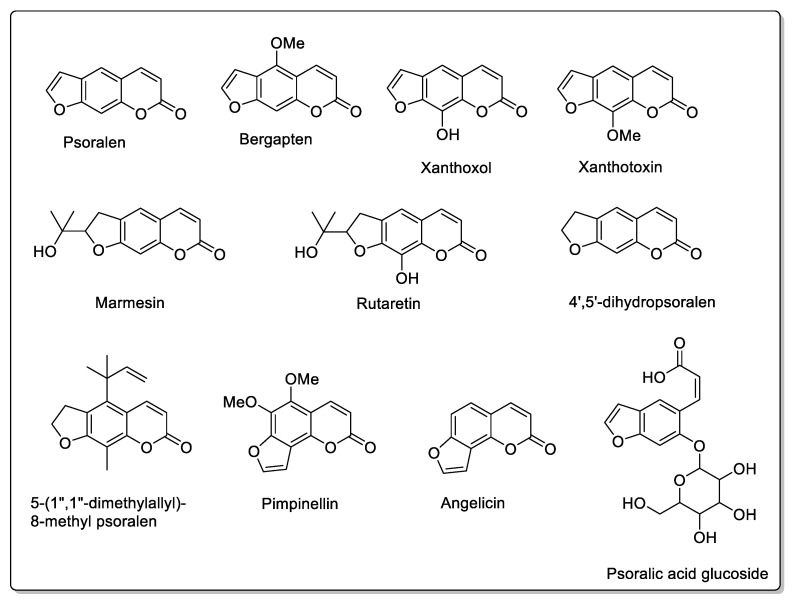
Some of the structures present in *ficus carica* that can induce drug interactions.

The fig tree (*ficus carica* L.) is a fruit tree in the *Moraceae* family. Ficus species contain alkaloids, terpenoids, and phenols. Alkaloids are of the indolizidine, chlorophenanthroindolizidine, septicine, and furoquinolines type; their role in the plant is to provide defensive properties [59,60,61,62,63]. Terpenoids include monoterpenoids, triterpenoids, sesquiterpenoids, steroids, and norisoprenoids, whose properties are somewhat distant from our subject, since they are antibacterial, insecticidal, and antiproliferative [64,65,66,67]. The phenolic compounds of ficus have been extensively studied and include, in addition to phenolic acids, flavonoids, coumarins, pyranocoumarins, furanocoumarins, and tannins, or phlobatannins. These derivatives have antioxidant, antimycobacterial, or nematicidal properties, which is not surprising given their structures, and they interact most with drugs [68,69,70,71]. *Ficus carica* L. is also known in traditional medicine for its numerous hepatoprotective, antifungal, anti-inflammatory, and hypoglycemic virtues. It has also been cited as a treatment for diabetes [72]. S. Igncimuthu et al. [73] studied the beneficial effect of *ficus carica* leaves on the glucose and lipid levels in type 2 diabetic rats. However, L. Wang et al. [74] who very recently documented the compounds and metabolites present at different levels of the plant, showed that the leaves contain psoralen and bergapten, in particular. Leaf decoctions could, therefore, be involved in drug interactions, given their composition. Fruits contain glycosylated quercetins and derivatives. Interactions with anticoagulants have also been reported. At the very least, the effect of ficus on glycemia could generate pharmacodynamic interactions, with synergistic and, therefore, additive and cumulative effects with antidiabetic treatments. The main furanocoumarins are, therefore, two linear isomers: psoralen and bergapten [69,70,75]. Another glycosylated compound of psoralen (psoralic acid glucoside) is also present in large quantities. Other linear furanocoumarins include marmesin, xanthotoxol [76], xanthotoxin, rutaretin [77], 4′,5′-dihydropsoralen, and 5-(1″,1″-dimethylallyl)-8-methylpsoralen [78]. In addition, there are two angular furanocoumarins: angelicin and pimpinellin [77] (Figure 9). Several of these compounds have been described in other Ficus species.

In addition to citrus fruits, apples, and figs, which are known for this type of drug interaction, other fruits are beginning to be widely studied, as they are being recommended for their benefits and, therefore, consumed without reservation; however, they may contain the constituents incriminated in citrus fruits.

### 2.3. Blackberry

**Figure 10:**
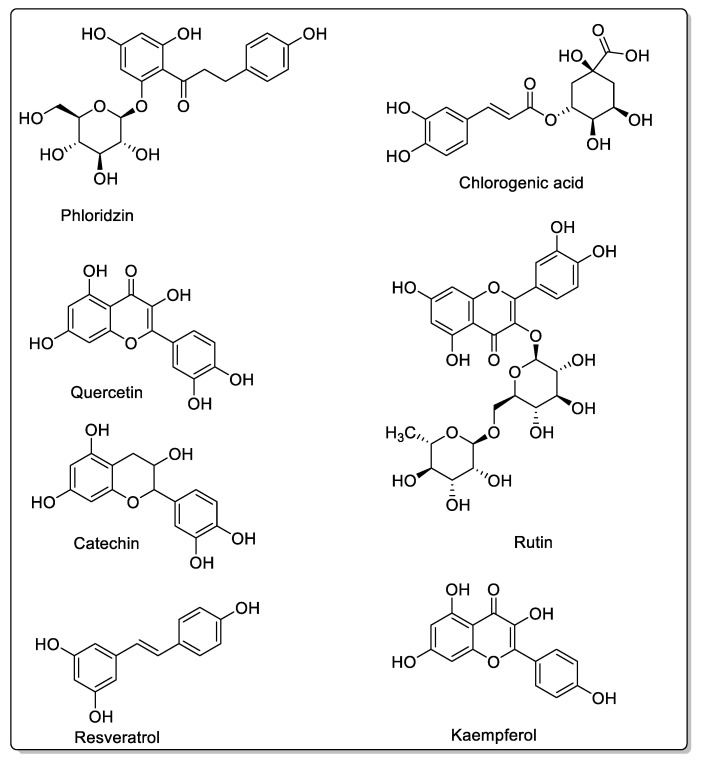
Structures present in blackberry.

The blackberry is a fruit belonging to the *Moraceae* family, of which there are many species, but the following three—*Morus alba*, *Morus nigra*, and *Morus rubra*—are the most studied for their medicinal properties [79]. This fruit is reputed to be beneficial in a wide range of pathologies and, therefore, has an extremely broad spectrum of action. Moreover, blackberries are readily available and marketed in a variety of forms. Numerous potential therapeutic applications have been reported, and, in juice form, blackberries are said to help boost immunity [80], as well as fight inflammation [81], chronic diseases [82], and liver problems [83]. These fruits contain compounds similar to those found in the fruits already mentioned, namely anthocyanins, phloridzin, and quercetin, as well as rutin, resveratrol, and chlorogenic acid (Figure 10). Different pathways leading to anthocyanins or flavonols [84] synthesize the widely present flavonoids. Blackberry juice has been reported by Y.C. Hou et al. [85] to inhibit CYP3A1 and OATP-B, as well as modulate CYP or even activate CYP3A1 and p-glycoprotein. They studied the impact on cyclosporine, an immunosuppressive drug used to prevent transplant rejection or treat rheumatoid arthritis [86]. Cyclosporine involves both CYP3A4 and P-glycoproteins (P-gp) during oral absorption, making it a drug of choice for testing potential interactions [87]. Rutin, quercetin, resveratrol, and kaempferol (Figure 10) have already been shown to modulate P-gp [88,89] and CYP3A4 [90,91,92]. The effects of these compounds on P-gp or CYP3A4 are sometimes inverse, sometimes inhibitory [93], and sometimes stimulatory [90], making it difficult to assess their overall effects. Synergistic effects may also be present, depending on the type of juice; however, in vivo, they reduce the oral bioavailability of cyclosporine in rats [94]. Indeed, YC Hou et al. [85] studied the effect of blackberry serum on cyclosporine pharmacokinetics in rats, and were able to demonstrate a decrease in cyclosporine absorption through activation of P-gp and CYP3A4. They incriminate quercetin and rutin in these interactions, although they are not present in very large quantities in blackberries, but advise against consumption of these fruits when taking drugs that are substrates of P-gp and/or CYP3A4. However, a study on human volunteers should be carried out to be sure, as we have seen with pomegranate juice that an in vitro interaction has not been confirmed in human clinical trials.

### 2.4. Apple

**Figure 11:**
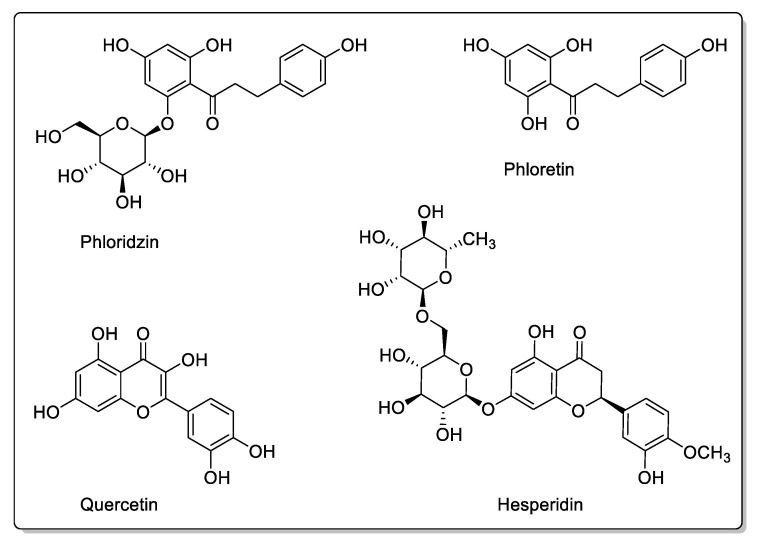
Flavonoids present in apple.

As mentioned in the introduction, apple juice ingestion has been shown to significantly reduce the absorption of certain drugs, notably fexofenadine, by prolonging the time required to reach peak plasma concentration [95]. Apple juice (*Malus domestica*, *Rosaceae* family) inhibited organic anion transporter polypeptide 2B1 (OATP2B1) in the intestine in a dose-dependent manner, without the need for repeated apple juice ingestion [95]. In their study, the authors did not mention the ingredients responsible for the observed effects, but others have shown that the flavonoids present in apple juice in varying quantities are phloridzin, phloretin, hesperidin, and quercetin (Figure 11). While furanocoumarins act primarily on CYPs, apple juice inhibits CYP1A1. Flavonoids act on membrane transporters, inhibiting drug absorption by OATP2B1 [21]. OATP1 and OATP3 are also inhibited, and the presence of polyphenols is involved [96,97]. However, various studies on other drugs not necessarily involving OATP2B1 have also shown interactions, and it appears that a plasma membrane monoamine transporter (PMAT/SLC29A4) may also be involved in these drug interactions at the level of ingestion. Yuasa H et al. showed that phloretin, quercetin, quercetin-3ß-d-glucoside, rutin, and phlorizin had inhibitory effects on PMAT [98]. Apple juice, on the other hand, generally has no modulatory effect on CYP3A [99].

### 2.5. Pomegranate

**Figure 12:**
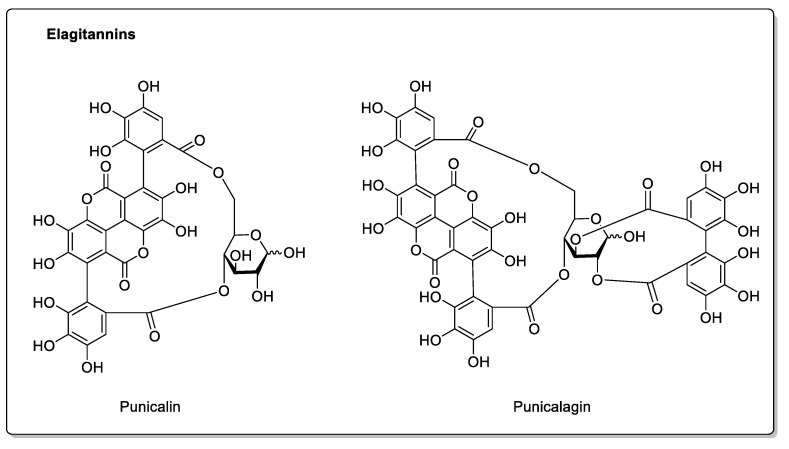
The elagitannins punicalin and punicalagin, specific to pomegranates.

Pomegranate (*Punica granatum* L. *Lythraceae* family) contains anthocyanins, which give it its characteristic color, and flavonoids, responsible for the antioxidant activity attributed to this fruit [100]. Other compounds, present in significant quantities and likely to present drug interactions, are hydroxycinnamic acids (Figure 4). The juice also contains catechins [101], proanthocyanidins, quercetin, and elagitannins (punicalin and punicalagin, specific to pomegranates, Figure 12) [102]. The much harder peel contains flavonoids, glycosylated and non-glycosylated flavonols, and glycosylated and non-glycosylated flavones. Pomegranate juice has been shown to inhibit CYP2C9 in vitro, and D.J. Greenblatt et al. [103] conducted a study on flurbiprofen in human volunteers; however, consuming this juice did not alter clearance of the drug. Similarly, another study on midazolam showed no significant change in the compound’s pharmacokinetics [104]. Despite the in vitro results, it appears that concomitant intake of pomegranate juice with the drugs tested does not affect their bioavailability and, therefore, has no in vivo inhibitory action on CYP3A4 [105]. On the other hand, the presence of polyphenols, and, therefore, antioxidants, effectively limits inflammation and oxidative stress [106]. To the best of our knowledge, no drug interactions have been demonstrated, and pomegranate is said to have beneficial effects. Unfortunately, this juice is not widely commercially available, unlike traditional juices.

### 2.6. Cranberry

**Figure 13:**
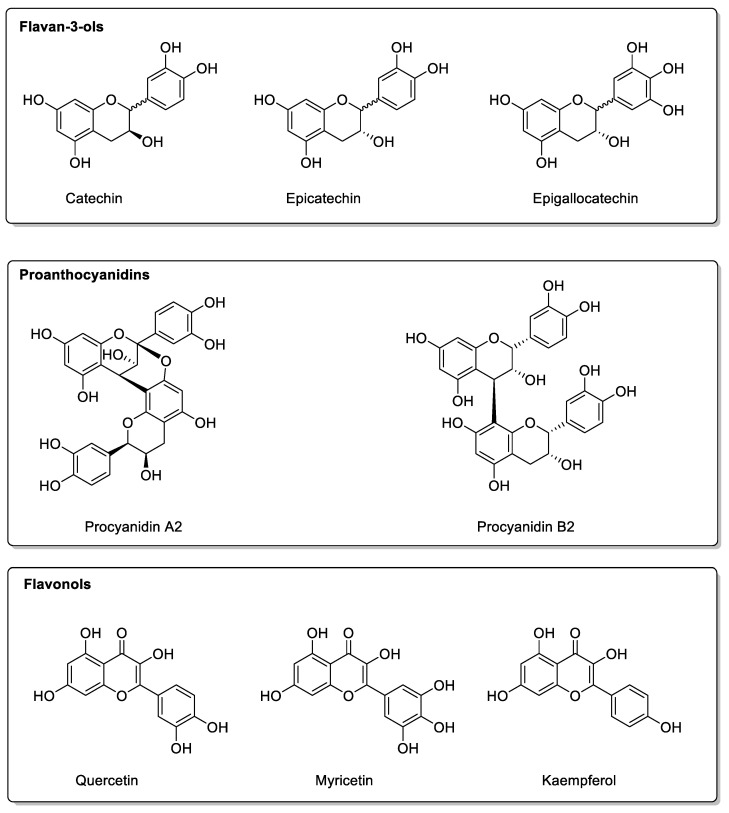
Structures present in cranberry.

In studies on human volunteers, cranberry juice (*Vaccinium macrocarpon*, *Ericaceae* family) did not alter drug pharmacokinetics. Warfarin, tizanidine, and midazolam were tested, and the CYPs involved were CYP2C9, CYP1A2, and CYP3A4, respectively [107,108,109,110]. As with pomegranate juice, its use in combination with flurbiprofen did not alter the CYP2C9-mediated clearance of flurbiprofen [111]. In the various studies, no drug interactions could be measured with cranberry juice, which was even used in combination with the triple antibiotic therapy of omeprazole, amoxicillin, and clarithromycin, although this study showed an improvement in the efficacy of these antibiotics against *H. pylori* bacteria in women [111]. Among the compounds responsible for these beneficial activities [112] are flavan-3-ol monomers and dimers; proanthocyanidins; anthocyanins; hydroxycinnamic acids, also found in citrus pulp; and a number of terpenes and flavonols [113]. As in pomegranate juice, flavan-3-ols of the epicatechin and catechin types, with traces of epigallocatechins and proanthocyanidins, are also present, which could perhaps prevent these drug interactions, since these compounds do not appear to be present in citrus fruits. Similarly, it has not been established with certainty which constituents are responsible for these drug interactions. The potentially interesting molecules present in cranberries are listed in Figure 13. Among the compounds most resistant to juice manufacturing processes such as pasteurization, while remaining sensitive to high temperatures, are flavonols and proanthocyanins, with anthocyanins being rather fragile constituents.

### 2.7. Blueberry

**Figure 14:**
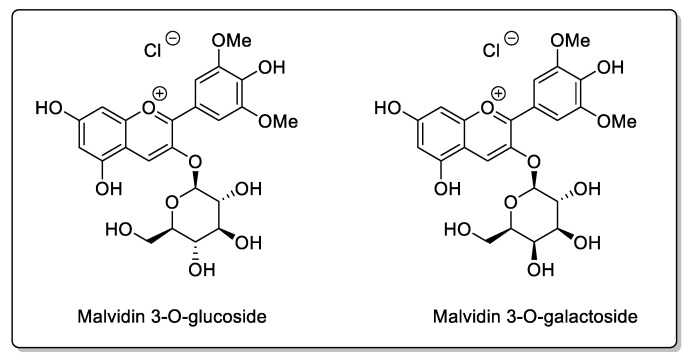
Anthocyanin derivatives present in greater quantities in blueberries.

Unlike many of the other fruits discussed herein, blueberry is beneficial in the treatment of certain diseases, and does not act on the same receptors as citrus juices [114]. In 2012, Greenblatt et al. [115] studied the potential interactions of blueberry juice with drugs. The anthocyanins and flavonoids present in blueberries have the same beneficial effects as those associated with cranberries [116,117] and account for a large proportion of the phenolic compounds present [118]. There are two main varieties of blueberry, *Vaccinium angustifolium* Aiton and *Vaccinium corymbosum* L., both from the *Ericaceae* family, but with different flavonoid contents [117]. Until 2012, the date of Greenblatt’s study [115], no clinical information was available on possible interactions with medical treatments. The CYP3A and CYP2C9 activities of blueberry juice were therefore assessed in humans, first in vitro with triazolam and buspirone for CYP3A evaluation, as well as with flurbiprofen as the indicator substrate for CYP2C9. Clinical trials were then conducted in volunteers, using buspirone and flurbiprofen as CYP3A and CYP2C9 substrates, respectively. In clinical studies, blueberry juice ingested by patients contained malvidin 3-glucoside and malvidin 3-galactoside (Figure 14), among anthocyanin derivatives present in greater quantities. In vitro, blueberry juice has been shown to inhibit the transformations of triazolam and buspirone, i.e., CYP3A substrates, as well as flurbiprofen; however, in this pharmacokinetic study, none of the clinical trials revealed any adverse effects, but the study was carried out on a very small number of individuals (nine men and three women). Blueberry juice may reduce the symptoms of certain diseases, particularly idiopathic arthritis; reduces levels of interleukin-1 alpha and beta; and increases levels of interleukin-1 receptor antagonists. Pang et al. [119] demonstrated in 2015 in their study on a larger clinical trial (201 patients with juvenile idiopathic arthritis) that blueberry juice could not only limit inflammation, but, at the same time, reduce the side effects of etanercept, thus improving the treatment of juvenile idiopathic arthritis with etanercept [119].

Fruits and their juices have been classified according to their potential drug interactions, starting with the *Rutaceae* family and ending with fruits whose in vivo interaction has not been proven. These fruits can, therefore, be consumed in conjunction with medical treatment without any proven risk of over- or under-dosing. The fruits mentioned in this review are listed in Table 1, together with their constituents and the biological interactions that may give rise to drug interactions.

Given the results of the studies presented here, covering several botanical families, we note that some of them may be involved in drug interactions, as they contain identical constituents (coumarins and furanocoumarins). However, although red fruits are considered analogous, they belong to botanical families that may be different. It seems that the *Ericaceae* or *Lythraceae* families contain additional chemical compounds more likely to have beneficial effects (cranberry; blueberry or pomegranate), and that no drug interactions have been proven for this family.

## 3. Conclusions

Studies on drug interactions are often carried out on fruit juices, as drugs are frequently taken with fruit juices to mask the taste or the astringency caused by some galenic forms. However, as some drugs are also taken with meals, it would be interesting to study potential interactions with the consumption of certain vegetables, especially if eaten raw. The literature on vegetables is still too sparse. It appears that, while citrus juices undoubtedly interfere with a large proportion of oral medications due to their inhibition of CYP3A4, this is not the case with some red fruits, including pomegranates, which could even have interesting synergistic effects, or reduce certain side effects. The impacts of the type of consumption—whole fruit or fruit juice—is important. The use of fresh or processed fruit also plays a role. Industrial fruit juices are made by pressing, and therefore include all constituents of the fruit, particularly in the case of citrus fruits: juice, pulp, and peel. These drug interactions are particularly important for the elderly, who often take several medications, increasing the risk of cross-interactions. Interactions are dose- and person-dependent, making prediction extremely difficult, hence the safety recommendation not to drink grapefruit juice while taking medication. Furthermore, no account is taken of variations in composition depending on where the fruit is grown and how it ripens. It would be interesting to test the various compounds present in these fruits alone or together, in order to envisage a natural constituent of this type that, added to the galenic form of the drugs, could increase their bioavailability or transport. This would make it possible to reduce useful doses and the associated side effects.

## Figures and Tables

**Table 1:** Summary of fruit CYP and drug transporter inhibitions.

Fruit	Family	Biological Interactions	Types of Constituents and Examples	Results in Terms of Drug Interactions
Grapefruit (*Citrus × paradise*)	*Rutaceae*	Irreversible inhibition of CYP3A4. Inhibition of P-gp.	Furanocoumarins: Psoralen; Bergaptol; Bergapten; Bergamottin; Epoxybergamottin; 6′,7′-dihydroxybergamottin; Oxypeucedanin.	Reduced absorption; decreased bioavailability.
		OATP and P-gp inhibitions.	Flavonoids: Narirutin; Hesperidin; Rutin; Narengenin; Naringin.	Decreased bioavailability.
Orange (*Valencia*)	*Rutaceae*	Incomplete inhibition of OATP1A2. Inhibition of OATP2B1.	No furanocoumarins. Flavonoids: Narirutin; Hesperidin; Phenolic acids: *p*-Coumaric acid; Ferulic acid.	No furanocoumarins. Fewer interactions generated.
Seville orange (*Citrus aurantium*)	*Rutaceae*	CYP3A4, CYP 2C9, and OATP inhibitions. P-gp not affected.	Furanocoumarins: Bergapten. Flavonoids: Narirutin; Naringin. Phenolic acids: *p*-Coumaric acid; Ferulic acid Caffeic acid; Chlorogenic acid.	Decreased bioavailability.
Lime (*Citrus aurantiifolia*, *Citrus latifolia*)	*Rutaceae*	CYP3A4, CYP2C9, and OATP2B1 inhibitions.	Same Furanocoumarins, flavonoids, and polyphenols as in grapefruits. Bergapten; Bergamottin; Oxypeucedanin; Narirutin; Naringin; Naringenin; Hesperetin; Rutin; Eriocitrin; Isonaringenin; Vicenin; Lucenin; *p*-Coumaric acid; Ferulic acid; Caffeic acid.	Reduced absorption; decreased bioavailability.
Pomelo (*Citrus grandis* L. *Osbeck* or *Citrus maxima*)	*Rutaceae*	Inhibition of CYP2C9, CYP3A4, and OATP2B1. P-gp not affected.	Coumarins: Rutin; Meranzin; Taddanone Flavonoids: Naringin; Hesperidin; Apigenin Tangeretin; Nobiletin; Taddanone Naringin; Epicatechin. Phenolic acids: *p*-Coumaric acid; Ferulic acid; Caffeic acid.	Reduced absorption; decreased bioavailability.
Fig (*Ficus carica* L.)	*Moraceae*	CYP3A4 inhibition.	Furanocoumarins Psoralen; Bergapten; Marmesin; Xanthotoxol; Xanthotoxin; Rutaretin; Angelicin; Pimpinellin.	Interactions have been reported with anticoagulant and antibacterial treatments.
Blackberry (*Morus alba*, *Morus nigra*, *Morus rubra*)	*Moraceae*	CYP3A4, CYP3A1, and OATP-B1 inhibitions. P-gp and CYP3A4 modulations.	Coumarins: Rutin. Flavonoids: Phloridzin; Quercetin; Kaempferol; Resveratrol. Phenolic acids: Chlorogenic acid. Anthocyanins.	Decreased bioavailability.
Apple (*Malus domestica*)	*Rosaceae*	PMAT/SLC29A4, CYP1A1, CYP2C9, OATP2B1, OATP1, and OATP3 inhibtions.	Flavonoids and polyphenols: Phloridzin; Phloretin; Hesperidin; Quercetin; Rutin.	Potential reduction in absorption. No effect on CYP3A.
Pomegranate (*Punica granatum* L.)	*Lythraceae*	CYP3A4 and CYP2C9 inhibitions in vitro.	Flavonoids: Catechins; Epicatechins; Quercetin. Phenolic acids: *p*-Coumaric acid; Ferulic acid; Caffeic acid; Chlorogenic acid. Proanthocyanidins. Eligatannins: Punicalin; Punicalagins.	Bioavailability unaffected in vivo.
Cranberry (*Vaccinium macrocarpon*)	*Ericaceae*	CYP3A4, CYP1A2, and CYP2C9 inhibitions in vitro.	Flavonols: Catechins; Epicatechin; Epigallocatechin; Quercetin; Kaempferol; Myricetin. Phenolic acids: *p*-Coumaric acid; Ferulic acid; Caffeic acid; Chlorogenic acid. Proanthocyanidins: Procyanidin A2; Procyanidin B2.	Bioavailability unaffected in vivo.
Blueberry (*Vaccinium angustifolium* Aiton and *Vaccinium corymbosum* L.)	*Ericaceae*	CYP3A inhibition in vitro.	Flavonols: Catechins; Epicatechin; Epigallocatechin; Quercetin; Kaempferol. Phenolic acid: *p*-Coumaric acid; Ferulic acidl Caffeic acid; Chlorogenic acid. Proanthocyanidins: Malvidin 3-glucoside; Malvidin 3-galactoside.	Bioavailability unaffected in vivo.

## Data Availability

Not applicable.

## Abbreviations

CYP	Cytochrome P450
OATP	Organic Anion-transporting Polypeptides
P-gp	P-glycoprotein
CNS	Central Nervous System
FW	Fresh Weight
HPLC	High-performance Liquid Chromatography
UV	Ultraviolet radiation
PMAT	Plasma Membrane monoamine Transporter
SLC29	Solute carrier 29 transporters
